# Asprosin induces vascular endothelial-to-mesenchymal transition in diabetic lower extremity peripheral artery disease

**DOI:** 10.1186/s12933-022-01457-0

**Published:** 2022-02-15

**Authors:** Mei You, Yushuang Liu, Bowen Wang, Li Li, Hexuan Zhang, Hongbo He, Qing Zhou, Tingbing Cao, Lijuan Wang, Zhigang Zhao, Zhiming Zhu, Peng Gao, Zhencheng Yan

**Affiliations:** Department of Hypertension and Endocrinology, Center for Hypertension and Metabolic Diseases, Daping Hospital, Army Medical University, Chongqing Institute of Hypertension, 10 Chang Jiang Zhi Lu, Yuzhong District, Chongqing, 400042 China

**Keywords:** Lower extremity peripheral artery disease, Type 2 diabetes mellitus, Asprosin, Endothelial-to-mesenchymal transition, TGF-β signaling pathway

## Abstract

**Background:**

Altered adipokine secretion in dysfunctional adipose tissue facilitates the development of atherosclerotic diseases including lower extremity peripheral artery disease (PAD). Asprosin is a recently identified adipokine and displays potent regulatory role in metabolism, but the relationship between asprosin and lower extremity PAD remains uninvestigated.

**Methods:**

33 type 2 diabetes mellitus (T2DM) patients (DM), 51 T2DM patients with PAD (DM + PAD) and 30 healthy normal control (NC) volunteers were recruited and the blood samples were collected for detecting the circulatory asprosin level and metabolomic screening. RNA sequencing was performed using the aorta tissues from the type 2 diabetic *db/db* mice and human umbilical vein endothelial cells (HUVECs) were treated with asprosin to determine its impact on the endothelial-to-mesenchymal transition (EndMT).

**Results:**

The circulating levels of asprosin in DM + PAD group were significantly higher than that of NC group and the DM group. Circulating asprosin level was remarkably negatively correlated with ankle-brachial index (ABI), even after adjusting for age, sex, body mass index (BMI) and other traditional risk factors of PAD. Logistic regression analysis revealed that asprosin is an independent risk factor for PAD and receiver-operator characteristic (ROC) curve determined a good sensitivity (74.5%) and specificity (74.6%) of asprosin to distinguish PAD. Data from metabolomics displayed a typical characteristics of de novo amino acid synthesis in collagen protein production by myofibroblasts in patients with PAD and activation of TGF-β signaling pathway appeared in the aortic tissue of *db/db* mice. Asprosin directly induces EndMT in HUVECs in a TGF-β-dependent manner as TGF-β signaling pathway inhibitor SB431542 erased the promotional effect of asprosin on EndMT.

**Conclusions:**

Elevated circulatory asprosin level is an independent risk factor of lower extremity PAD and might serve as a diagnostic marker. Mechanistically, asprosin directly induces EndMT that participates in vascular injury via activation of TGF-β signaling pathway.

*Trial registration* This trial was registered at clinicaltrials.gov as NCT05068895

**Supplementary Information:**

The online version contains supplementary material available at 10.1186/s12933-022-01457-0.

## Background

Lower extremity peripheral artery disease (PAD) is atherosclerotic occlusive disease of the peripheral arteries supplying the legs [1]. According to a recent systematic review, 238 million people worldwide suffered from PAD that has been recognized as a global health issue contributing to increased disability-adjusted life-years, years of life lost, and years lived with disability [2]. Patients with PAD not only endure a lowered quality of life resulting from intermittent claudication or atypical ischemic leg pain, but also possess a higher mortality rate as PAD is also a strong predictor of future cardiovascular events, such as myocardial infarction and stroke [3, 4]. In addition to age, sex and genetic background, PAD also shares similar risk factors with other atherosclerotic diseases, such as smoking and components of metabolic syndrome that include obesity, diabetes mellitus, dyslipidemia, and hypertension [5]. Thus, currently, in addition to lifestyle and drugs that against the above-mentioned risk factors, endovascular intervention or surgery are mainly applied in the treatment of PAD and needless to say, specific diagnostic and treatment approaches for PAD are urgently needed [3].

Excess adipose tissue accumulation is a hallmark of the metabolic syndrome that facilitates the development of atherosclerotic diseases including PAD [6]. Acting as a newly identified endocrinological organ, adipose tissue secretes a series of adipokines, such as leptin, adiponectin, fibroblast growth factor 21 (FGF21), etc. to participate in the regulation of the metabolism and function of cardiovascular system [7]. Altered adipokine synthesis and secretion in dysfunctional adipose tissue contributes to a spectrum of metabolic atherosclerotic arterial diseases [8]. Asprosin, the C-terminal cleavage product of profibrillin, is a recently identified adipokine that displays potent regulatory role in metabolism [9]. In addition to stimulating hepatic gluconeogenesis that increases blood insulin and glucose levels, asprosin also directly promotes the apoptosis of pancreatic β-cells [10]. Accordingly, elevated plasma asprosin levels are detected in diabetic patients, suggesting a possible promotional effect of asprosin in diabetic vascular damage [11, 12]. However, the relationship between asprosin and lower extremity PAD in diabetic status has not been investigated.

Endothelial dysfunction, characterized by lowered flow-mediated dilation (FMD), has long been recognized as an independent predictor of cardiovascular risk in patients with PAD and adds to the predictive value of ankle-brachial index (ABI), the conventional parameter that widely used to diagnosis of PAD [13]. Mechanistically, increased level of advanced glycation end-products (AGEs) due to hyperglycemia induces reactive oxygen species (ROS) production that inactivates endothelium-derived nitric oxide (NO) and uncoupled endothelial NO synthase (eNOS) activity, resulting in inflammation that contribute to the vascular damage [14]. It is worth noting that compared with coronary atherosclerotic plagues which contain more lipid, most of the lower extremity arteries in patients with PAD display medial calcification that leads to stiffening of the peripheral arteries [15]. Vascular calcification is an active process that due to the transdifferentiation of vascular cells, in which endothelial-to-mesenchymal transition (EndMT) mainly affects endothelial cells (ECs) [16]. During the process of EndMT, the tight cell–cell junctions between ECs are disrupted, their cobblestone-like well-structured appearance switches to spindle-shaped, fibroblast-like one, in which TGF-β signaling pathway plays a central role [17, 18]. Although EndMT is considered as a critical mechanism underlying endothelial dysfunction, whether EndMT participates in lower extremity PAD and whether asprosin plays a role in EndMT remains unknown.

In this study, patients with PAD were recruited and we confirmed that elevated plasma asprosin level was independently associated to various parameters of PAD. Furthermore, through metabolic omics screening and cell experiments, we confirmed that EndMT was an important process related to PAD and revealed a previously unrecognized molecular mechanism underlying the promotional effect of asprosin on EndMT, thus updated the understanding on the vascular injury in PAD and provided a new target for treatment and intervention of PAD in future clinical practice.

## Methods

### Study subjects

This cross-sectional single-center study was approved by the Ethics Committee of Army Medical Center of PLA. The experimental procedures were conducted according to the Declaration of Helsinki. Informed consent was obtained from each participant. The protocol was registered in ClinicalTrials.gov (Identifier: NCT05068895). PASS 15.0 software (NCSS, LLC) was used to calculate the sample size. The expected area under curve (AUC) of asprosin was about 0.7 [11, 19], and the calculated size is N = 82 for this study.

33 type 2 diabetes mellitus (T2DM) patients (DM) and 51 T2DM patients with PAD (DM + PAD) were recruited from the Department of Hypertension and Endocrinology, Daping Hospital, Army Medical University, Chongqing, China, from June to October 2021. All patients were confirmed to be diagnosed with T2DM on the basis of the American Diabetes Association criteria with fasting plasma glucose (FPG) ≥ 7.0 mmol/l, or hemoglobin A1c (HbA1c) ≥ 6.5% or oral glucose tolerance test (OGTT) 2 h post-load plasma glucose ≥ 11.1 mmol/l or self-reported medical history [20]. Besides, the diagnosis of T2DM with PAD was based on standard criteria recommended by American Heart Association [3] with a resting ABI ≤ 0.9 or imaging method including duplex ultrasound (DUS), computed tomography angiography (CTA) or digital subtraction angiography (DSA) showed stenosis or thrombosis existence in lower extremity peripheral arteries.

The following were the main exclusion criteria: (1) type 1 diabetes and other special types of diabetes, (2) type 2 diabetes with acute diabetic complications such as diabetic ketoacidosis, hyperosmolar hyperglycemic status and diabetic lactic acidosis, (3) acute infection at the time of evaluation, (4) cardiovascular or cerebrovascular disease, liver or renal dysfunction, tumors, autoimmune disease or mental disease, (5) a history of lower extremity amputations due to diabetes, (6) alcohol abuse or pregnancy. Additionally, we enrolled 30 additional age-, sex-, body mass index (BMI)- and waist circumference- matched healthy volunteers as normal controls (NC) with normal FPG and HbA1c.

### Clinical and routine laboratory measurements

Demographic characteristics, previous history of smoking and medication history (including anti-diabetic medications, statins, antiplatelets treatment and anti-hypertensive drugs) were collected through the electronic medical record. Physical examination included measurements of height, weight and waist circumference, and BMI (kg/m^2^) was calculated as weight divided by the square of height in meters. Office blood pressure (BP) was measured on both arms in the setting position after 10 min of resting for three times and the average value was calculated. ABI and toe-brachial index (TBI) measurement was performed by trained assessors from both sides, and the lower one was shown in our study. ABI was obtained by comparing the higher brachial systolic pressure with the higher pressure at the ankle (either the dorsal pedal [DP] or posterior tibial [PT] artery). This can be performed with a hand-held Doppler probe (Huntleigh, Cardiff, CF24 5HN, UK). Toe pressures were obtained by placing cuffs around each toe with a digital flow sensor beyond the cuff. The same as ABI, TBI was obtained by comparing the higher brachial systolic blood pressure with the higher pressure from the toe artery [21]. Blood samples were obtained in the morning after an overnight fast, and some samples were used for the measurement of FPG, HbA1c, high-sensitivity C-reactive protein (hs-CRP) and lipid profiles, including total cholesterol (TC), triglyceride (TG), high-density lipoprotein cholesterol (HDL-C) and low-density lipoprotein cholesterol (LDL-C) in the Endocrinology & Metabolism laboratory of Daping Hospital. Other samples were collected in a centrifuge tube and allowed to stand at room temperature for 1 h for coagulation and stratification. Then the samples were centrifuged at 3000 rpm for 10 min at room temperature and the supernatant was transferred to a clean centrifuge tube to centrifuge at 12000 rpm for 10 min at 4 ℃, and the plasma was aliquoted and stored at − 80 ℃ for further study. When the samples were collected enough (15 samples in each group), 200 μl of each sample were shipped by dry ice to the Shanghai Biotree biotech Company to perform liquid chromatography–mass spectrometry (LC–MS). When the recruitment of participant was stopped, serum asprosin concentrations were measured using a human Asprosin ELISA kit (ab275108, Abcam) following the instructions of the manufacturer.

### LC–MS-based nontargeted metabolomic approach

For extraction of metabolites, 50 μl of sample was transferred to an EP tube. After the addition of 200 μl of extract solution (acetonitrile: methanol = 1: 1, containing isotopically-labelled internal standard mixture), the samples were vortexed for 30 s, sonicated for 10 min in ice-water bath, and incubated for 1 h at −40 ℃ to precipitate proteins. Then the sample was centrifuged at 12,000 rpm for 15 min at 4 ℃ The resulting supernatant was transferred to a fresh glass vial for analysis. The quality control (QC) sample was prepared by mixing an equal aliquot of the supernatants from all samples. And then, LC–MS/MS analyses were performed using an UHPLC system (Vanquish, Thermo Fisher Scientific) with a UPLC BEH Amide column (2.1 mm × 100 mm, 1.7 μm) coupled to Q Exactive HFX mass spectrometer (Orbitrap MS, Thermo). The mobile phase consisted of 25 mmol/l ammonium acetate and 25 ammonia hydroxides in water (pH 9.75) (A) and acetonitrile (B). The auto-sampler temperature was 4 ℃, and the injection volume was 3 μl. The QE HFX mass spectrometer was used for its ability to acquire MS/MS spectra on information-dependent acquisition (IDA) mode in the control of the acquisition software (Xcalibur, Thermo). The raw data were converted to the mzXML format using ProteoWizard and processed with an in-house program, which was developed using R and based on XCMS, for peak detection, extraction, alignment, and integration. Then an in-house MS2 database (BiotreeDB) was applied in metabolite annotation. The cutoff value for annotation was set at 0.3.

### Animal experiments

All experimental procedures were performed in adherence to the NIH Guide for the Care and Use of Laboratory Animals and in accordance with protocols approved by the institutional animal care and research advisory committee at Daping hospital, Army Medical University. The *db/db* mice on C57BLKS/J background (000697), a model of type 2 diabetes in which leptin receptors are deficient, and their nondiabetic heterozygote littermates *db/m* mice, were purchased from Jackson Laboratory (Bar Harbor, ME). All mice were housed in cages at a controlled temperature (22 ± 1 °C) and relative humidity (55 ± 5%) in a 12-h light/12-h dark cycle. They were supplied with standard laboratory chow and tap water ad libitum. 12-week-old male mice were used to collect tissue samples. At the end of the treatment period, mice were sacrificed after fasting for 14 h. The aorta tissues were collected and quickly frozen in liquid nitrogen for further analysis.

### RNA sequencing (RNA-seq)

Total RNA was extracted from the tissues using Trizol (Invitrogen, Carlsbad, CA, USA) according to manual instruction. Subsequently, total RNA was qualified and quantified using a Nano Drop and Agilent 2100 bioanalyzer (Thermo Fisher Scientific, MA, USA). The RNA library construction and subsequent RNA sequencing were performed by BGI-Shenzhen, China. First-strand cDNA was generated using random hexamer-primed reverse transcription, followed by a second-strand cDNA synthesis. afterwards, A-Tailing Mix and RNA Index Adapters were added by incubating to end repair. The cDNA fragments obtained from previous step were amplified by PCR, and products were purified by Ampure XP Beads, then dissolved in EB solution. The product was validated on the Agilent Technologies 2100 bioanalyzer for quality control. The double stranded PCR products from previous step were heated denatured and circularized by the splint oligo sequence to get the final library. The single strand circle DNA (ssCir DNA) was formatted as the final library. The final library was amplified with phi29 to make DNA nanoball (DNB) which had more than 300 copies of one molecular, DNBs were loaded into the patterned nanoarray and single end 50 bases reads were generated on BGIseq500 platform (BGI-Shenzhen, China).

The sequencing data was filtered with SOAPnuke (v1.5.2) by and the clean reads were mapped to the reference genome using HISAT2 (v2.0.4). Bowtie2 (v2.2.5) was applied to align the clean reads to the reference coding gene set, then expression level of gene was calculated by RSEM (v1.2.12). The heatmap was drawn by pheatmap (v1.0.8) according to the gene expression in different samples. Essentially, differential expression analysis was performed using the DESeq2(v1.4.5) with false discovery rate (FDR)-adjusted *P*-value (q-value) ≤ 0.05. To take insight to the change of phenotype, GO (http://www.geneontology.org/) and KEGG (https://www.kegg.jp/) enrichment analysis of annotated different expressed gene was performed by Phyper (https://en.wikipedia.org/wiki/Hypergeometric_distribution) based on Hypergeometric test. The significant levels of terms and pathways were corrected by FDR-adjusted *P*-value (q-value), with a rigorous threshold (q ≤ 0.05) by Bonferroni. All analysis were performed on the Dr. Tom analysis system constructed by BGI-Shenzhen, China.

### Cell culture

Primary human umbilical vein endothelial cells (HUVECs) were purchased from Procell, China. Cells between four to seven passages were cultured in endothelial growth medium (CM-H082, Procell) containing 5% fetal bovine serum (FBS) and 1% penicillin/streptomycin with all the growth factor supplied at 37℃ with 5% CO_2_. At approximately 80% confluence, the culture medium was changed to a serum-free DMEM (GIBCO, Rockville, MD, USA) medium for 24 h before the cells were used for further experiments.

### In vitro* induction of EndMT*

To examine the effect of asprosin on the phenotypic transition in HUVECs, cells were treated with PBS (vehicle) or 50 μmol/l asprosin (HY-P7612, MCE) for 24 h. In order to further examine the effect of TGF-β inhibition on asprosin-induced EndMT, cells were treated with DMSO (vehicle) or 10 μmol/l SB431542 (HY-10431, MCE), a specific TGF-β type I receptor inhibitor, for the last 8 h.

### Immunofluorescent staining

For immunofluorescent microscopy, HUVECs were labeled with primary antibodies overnight, followed by incubation with a suitable fluorophore-conjugated secondary antibody for 1 h. Specifically, goat anti-mouse-CD31 antibody (MA3100, 1:200; Invitrogen), goat anti-rabbit-alpha smooth muscle actin (α-SMA) antibody (ab124964, 1:300; Abcam) were used to stain the makers of HUVECs and VSMCs, respectively. Immunofluorescent images were obtained using a confocal microscope (A1R HD25, Nikon, Tokyo, Japan) with NIS-Elements BR software (version 3.2. Nikon).

### Quantitative PCR

Total RNA was isolated from HUVECs using TRIzol (15,596,026, Invitrogen™). First strand cDNA was synthesized using random primers and EvoScript Universal cDNA Master (Roche, Germany). PCR reactions were carried out with the manufacturer (Light Cycler 96, Roche), using the FastStart Essential DNA Green Master (Roche, Germany). Light Cycler analysis software (Life Technologies, Norwalk, CT) was used to determine crossing points using the second derivative method. Data were normalized to housekeeping genes (β-actin). Details of the primer sequences used are presented in Additional file 1: Table S1.

### Western blot

First, HUVECs were lysed in RIPA lysis buffer (65 mM Tris–HCl pH 7.5, 150 mM NaCl, 1 mM EDTA, 1% Nonidet P-40, 0.5% sodium deoxycholate and 0.1% SDS), protease inhibitor cocktail tablets (04693132001; Roche) and phosphatase inhibitor tablets (4906837001; Roche). The protein concentration was determined using the BCA Protein Assay Kit (23225; Thermo Fisher Scientific). Proteins were separated on 10% SDS–PAGE gels and then transferred to PVDF membranes (IPVH00010; Millipore). The membranes were blocked for 1 h at room temperature in Tris-buffered saline and 0.1% Tween-20 (TBST) containing 5% skim milk and then were incubated with primary antibodies in the same buffer at 4 °C overnight as followed: Smad 2/3 (sc-133098, Santa Cruz), p-Smad2/3 (sc-11769, Santa Cruz), TGF-β1 (ab215715, Abcam), CD31 (sc-376764, Santa Cruz), VWF (sc-14014, Santa Cruz), NOS3 (sc-136977, Santa Cruz), α-SMA (ab7817, Abcam), TAGLN (ab14106, Abcam), collagen I (ab88147, Abcam), CTGF (sc-34772, Santa Cruz), β-actin (66009-1-Ig, Proteintech). After washes and incubation with the appropriate horse radish peroxidase-conjugated secondary antibody (Santa Cruz Biotechnology), the immune complexes were visualized using a chemiluminescence reagent. Western blot results were densitometrically quantified with Quantity One software (Bio-Rad), and the intensity values were normalized to β-actin.

### Statistical analysis

The continuous variables were expressed as means (mean ± standard deviation [SD]), or as medians (interquartile range [IQR]) based on the distribution, which is determined by the Shapiro–Wilk test. The categorical variables were expressed as n (%). The differences between groups were analyzed by ANOVA test (continuous variables with normal distribution) or Kruskal–Wallis H test (continuous variables with skewed distribution) or chi-squared test (categorical variables). Spearman’s correlative analysis was used to analyze the correlations of circulating asprosin levels with ABI and TBI. Multiple linear regression analysis was conducted to further evaluate the relationship between circulating asprosin levels and ABI/TBI in different models. Multiple logistic regression analysis was used to determine the effect on circulating asprosin levels the risk of PAD. The area under the receiver operating characteristic (ROC) curve was calculated to test the discrimination of PAD. *P* < 0.05 was set as statistically significant. Graphs were created using Prism 8.0 (GraphPad Software). All the statistical analyses about clinical data were performed using SPSS 26.0 software (SPSS Inc., Chicago, USA), while all the statistical analyses about cells were performed with GraphPad Prism.

## Results

### Characteristics of participants in different groups

The clinical characteristics of recruited patients, including DM and DM + PAD, as well as NC, are shown in Table 1. The sex ratio, age, BMI and waist circumference were similar among the three groups. In the diabetic patients, the smoking status, duration of diabetes and antidiabetic drug use including oral hypoglycemic agents and insulin treatment did not show any difference between the two groups. While the DM + PAD group displayed a higher rate of medication including statins, antiplatelet drugs and antihypertensives than the DM group.

**Table 1:** Demographic and clinical data of subjects in different groups

Variables	NC (N = 30)	DM (N = 33)	DM + PAD (N = 51)
Male (%)	53.3	54.5	62.7
Age (years)	60.63 ± 8.08	60.85 ± 13.91	64.53 ± 9.98
BMI (kg/m^2^)	25.76 ± 2.72	25.21 ± 2.40	24.85 ± 2.95
Waist circumference (cm)	86.83 ± 5.98	86.55 ± 7.31	86.49 ± 8.86
Smoking, current/past (%)	26.7	45.5	66.7^a^
Duration of DM (years)	–	8(3–14)	13(4.5–20)
Oral hypoglycemic agents (%)	–	97.0	98.0
Insulin (%)	–	90.9	82.4
Statins (%)	–	60.6	94.1^b^
Antiplatelet drugs (%)	–	21.2	92.2^b^
Antihypertensives (%)	26.7	15.2	66.7^ab^
ABI	1.08 ± 0.05	1.09 ± 0.08	0.67 ± 0.29^ab^
TBI	0.79 (0.69–0.85)	0.70 (0.69–0.80)	0.35 (0.24–0.56)^ab^
SBP (mmHg)	118.20 ± 9.00	134.03 ± 19.87^a^	141.35 ± 23.75^a^
DBP (mmHg)	91.63 ± 9.13	74.00 ± 10.96^a^	71.76 ± 11.66^a^
HbA1c (%)	5.71 (5.30–6.01)	8.90 (7.60–11.60)^a^	8.50 (7.05–9.95)^a^
FPG (mmol/L)	4.95 (4.51–5.28)	8.80 (6.90–11.70)^a^	7.80 (6.60–9.25)^a^
TC (mmol/L)	5.03 (4.13–5.53)	4.61 (3.95–5.19)	3.93 (3.29–4.93)^a^
TG (mmol/L)	0.98 (0.71–1.34)	1.40 (1.06–2.15)^a^	1.37 (1.15–1.86)^a^
HDL-C (mmol/L)	1.52 (1.29–1.76)	1.12 (0.96–1.28)^a^	0.92 (0.81–1.17)^a^
LDL-C (mmol/L)	3.01 (2.67–3.39)	2.87 (2.54–3.38)	2.53 (2.05–3.15)^a^
hs-CRP (mg/L)	0.47 (0.16–1.03)	0.50 (0.50–1.22)	7.19 (0.53–21.75)^ab^
Asprosin (ng/mL)	158.50 (139.18–180.75)	173.72 (158.75–213.57)	218.14 (184.23–305.65)^ab^

Value are proportions, and means (standard deviations) or medians (interquartile range)

*BMI* body mass index, *ABI* ankle-brachial index, *TBI* toe-brachial index, *SBP* systolic blood pressure, *DBP* diastolic blood pressure, *HbA1c* hemoglobin A1c, *FPG* fasting plasma glucose, *TC* total cholesterol, *TG* triglyceride, *HDL-C* high-density lipoprotein cholesterol, *LDL-C* low-density lipoprotein cholesterol, *hs-CRP* high-sensitivity C-reactive protein

^a^*P* < 0.05 compared with NC group; ^b^*P* < 0.05 compared with DM group

**Fig. 1:**
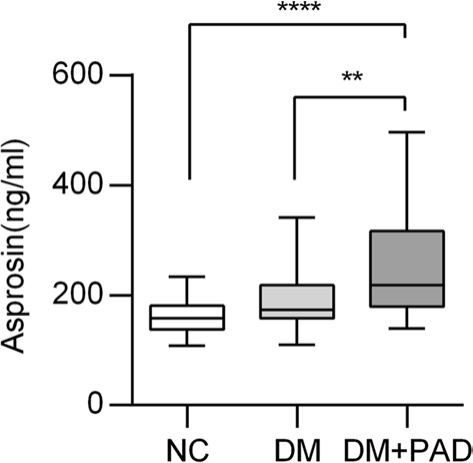
The circulating asprosin levels in NC, DM and DM + PAD group. ***P < 0.001, **P < 0.01, Data are medians with max and min values

Compared with NC group, both DM and DM + PAD groups displayed significantly higher systolic blood pressure (SBP), HbA1c, FPG, TG and lower diastolic blood pressure (DBP) and HDL-C. In addition, when further compared with DM group, patients in DM + PAD group showed higher hs-CRP and lower ABI and TBI. However, there was no significant difference of SBP, DBP, HbA1c, FPG, TC, TG, HDL-C and LDL-C between the DM and DM + PAD groups, suggesting that the occurrence of PAD would not be related to blood pressure, blood glucose or blood lipid levels in these patients. Besides, the circulating levels of asprosin in DM + PAD group were significantly higher than that of NC group and the DM group, while there was no significant difference between these two non-PAD groups (Fig. 1).

### Correlation of circulating asprosin levels with ABI and TBI

**Fig. 2:**
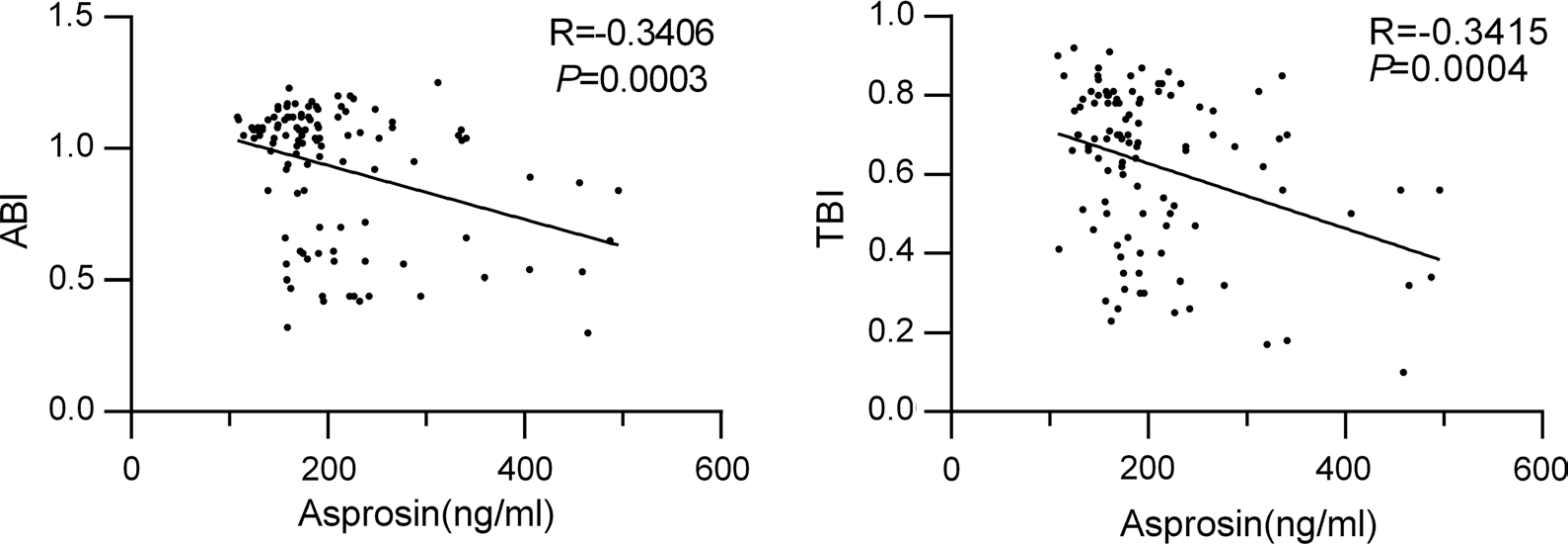
Correlation between circulating asprosin levels and ABI/TBI in all subjects

To further investigate the potential relationship between circulating asprosin levels and PAD, spearman analysis was performed. As shown in Fig. 2, in all patients and normal controls, circulating asprosin level was remarkably negatively correlated with ABI, the first-line noninvasive diagnostic method for PAD (r = − 0.3406, *P* < 0.001); or TBI, the alternative physiological testing for PAD (r = − 0.3415, *P* < 0.001). When further analyzed in multiple linear regression analysis models (Table 2), circulating asprosin level was also closely associated with ABI/TBI even after adjusting for age, sex and BMI (*P* < 0.001, model 2). Next, we further added more variables which considered as traditional risk factors of PAD, including smoking status, duration of diabetes, SBP, DBP, HbA1c, TC, HDL-C and LDL-C, and asprosin remained significantly correlated with ABI (*P* = 0.049, model 3), suggesting a close relationship between asprosin and ABI. While taking the same variables into account, asprosin did not display significant co-relationship with TBI (*P* = 0.054, model 3).

**Table 2:** Correlation of circulating asprosin levels with ABI and TBI in all subjects

Models	Independent variable	β Coefficient	95% confidence interval	*P*-value
ABI				
Model 1	Asprosin (100 ng/ml increase)	− 0.128	− 0.189 to − 0.068	**< 0.001**
Model 2	Asprosin (100 ng/ml increase)	− 0.124	− 0.183 to − 0.064	**< 0.001**
Model 3	Asprosin (100 ng/ml increase)	− 0.060	− 0.120 to − 0.000	**0.049**
TBI				
Model 1	Asprosin (100 ng/ml increase)	− 0.104	− 0.158 to − 0.051	** < 0.001**
Model 2	Asprosin (100 ng/ml increase)	− 0.101	− 0.151 to − 0.050	** < 0.001**
Model 3	Asprosin (100 ng/ml increase)	− 0.049	− 0.099 to − 0.001	0.054

The bold values indicate the *P*-values which were < 0.05

Model 1: crude, Model 2: adjusted for age, sex and BMI, Model 3: Model 2 plus smoking status, duration of diabetes, SBP, DBP, HbA1c, TC, HDL-C and LDL-C

*ABI* ankle-brachial index, *TBI* toe-brachial index

### Correlation of circulating asprosin levels with the risk of PAD

**Fig. 3:**
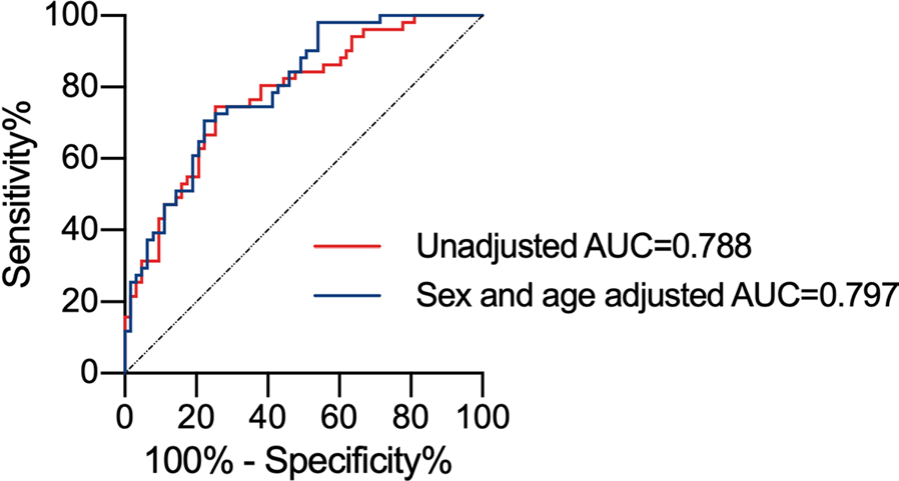
Receiver operating characteristic (ROC) curves for asprosin in all subjects. The curve shows the capability of asprosin measurement for determination of PAD. *AUC* area under the curve

Furtherly, the association of circulating asprosin levels with PAD was determined using multiple logistic regression analysis (Table 3). Before adjusting (model 1), circulating asprosin level showed significantly increased odds ratios (ORs) for PAD (OR = 4.882, *P* < 0.001). After adjusting for age, sex, and BMI (model 2), increased ORs also existed (OR = 5.473, *P* < 0.001). Even after further adjustment for smoking status, duration of diabetes, SBP, DBP, HbA1c, TC, HDL-C and LDL-C in model 3, asprosin still showed significantly increased ORs for PAD (OR = 3.944, *P* = 0.002), which suggesting high circulating asprosin level was associated with an increased risk of PAD independent of traditional risk factors. Moreover, ROC curve was generated to evaluate the discriminative capability of asprosin for determination of PAD. In all subjects, the findings indicated that a cut-off value of 188.70 ng/ml had a good sensitivity (74.5%) and specificity (74.6%) to distinguish PAD from non-PAD (Area under curve [95% CI] 0.778 [0.694–0.863], *P* < 0.001). And AUC adjusted for sex and age was 0.797 (95% CI 0.717–0.876), *P* < 0.001 (Fig. 3).

**Table 3:** Association of circulating asprosin levels with PAD by logistic regression analyses

Models	Independent variable	Odds ratio	95% confidence interval	*P*-value
Model 1	Asprosin (100 ng/mL increase)	4.882	2.242 to 10.633	** < 0.001**
Model 2	Asprosin (100 ng/mL increase)	5.473	2.368 to 12.649	**< 0.001**
Model 3	Asprosin (100 ng/mL increase)	3.944	1.656 to 9.393	**0.002**

Model 1: crude, Model 2: adjusted for age, sex and BMI, Model 3: Model 2 plus smoking status, duration of diabetes, SBP, DBP, HbA1c, TC, HDL-C and LDL-C. The bold values indicate the *P*-values which were < 0.05

### Metabolomic results reveals a causal relationship between TGF-β signaling pathway and lower extremity PAD

**Fig. 4:**
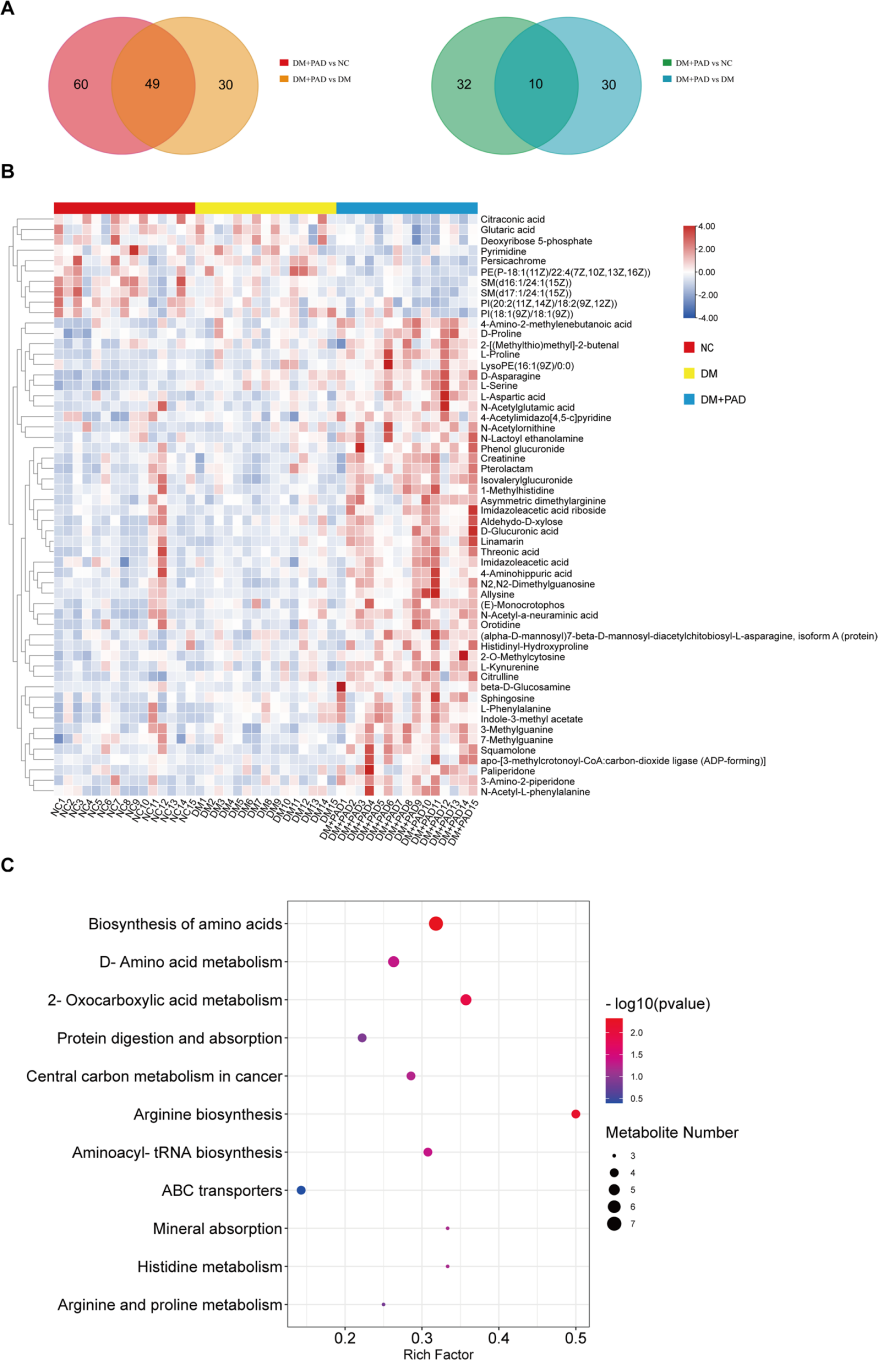
The metabolomic analysis on serums from participants. **A** The Venn diagram showing the numbers of the upregulated (left) and down-regulated (right) metabolites in DM + PAD group compared to either DM or NC group. **B** The heatmap of the 59 differentially expressed metabolites that exist simultaneously in DM + PAD group compared to both DM and NC group. **C** KEGG pathway analysis of the 59 differentially expressed metabolites

To further investigate the possible mechanism accounting for the lower extremity vascular injury in diabetic patients, we screened the serum samples from the three groups of participates for metabolomic analysis. Using One-way ANOVA, 31 differentially expressed metabolites were screened out and KEGG analysis showed that these metabolites were mainly enriched in biosynthesis of amino acids (Additional file 1: Fig. S1A, Additional file 2: Table S2). Through KEGG analysis, these metabolites were mainly enriched in biosynthesis of amino acids, especially the synthesis of proline from glutamic acid, arginine, or ornithine (Additional file 1: Fig. S1B). Next, as the clinical data imply that asprosin correlates with ABI independent of FPG level, we picked out 59 metabolites that simultaneously differentiated expressed in DM + PAD group compared to either DM or NC group, respectively (Fig. 4A, B, Additional file 3: Table S3). These metabolites represent the effect of PAD independent of diabetic status. The results of KEGG analysis confirmed that these metabolites were also mainly enriched in biosynthesis of amino acids again (Fig. 4C), showing a typical characteristics of de novo amino acid synthesis in collagen protein production by myofibroblasts in which TGF-β signaling pathway might plays a critical role [22]. We also screened out 4 metabolites that were simultaneously differentially expressed in both DM versus NC group and DM + PAD versus DM group (Additional file 1: Fig. S2A, Additional file 4: Table S4). These 4 metabolites represent the effect of diabetic status and KEGG analysis did not show a clear enrichment pathway (Additional file 1: Fig. S2B). Therefore, these results imply that there might exist a causal relationship between TGF-β signaling pathway and vascular injury in lower extremity PAD.

### Activation of TGF-β signaling pathway appeared in the aortic tissue of type 2 diabetic mice

**Fig. 5:**
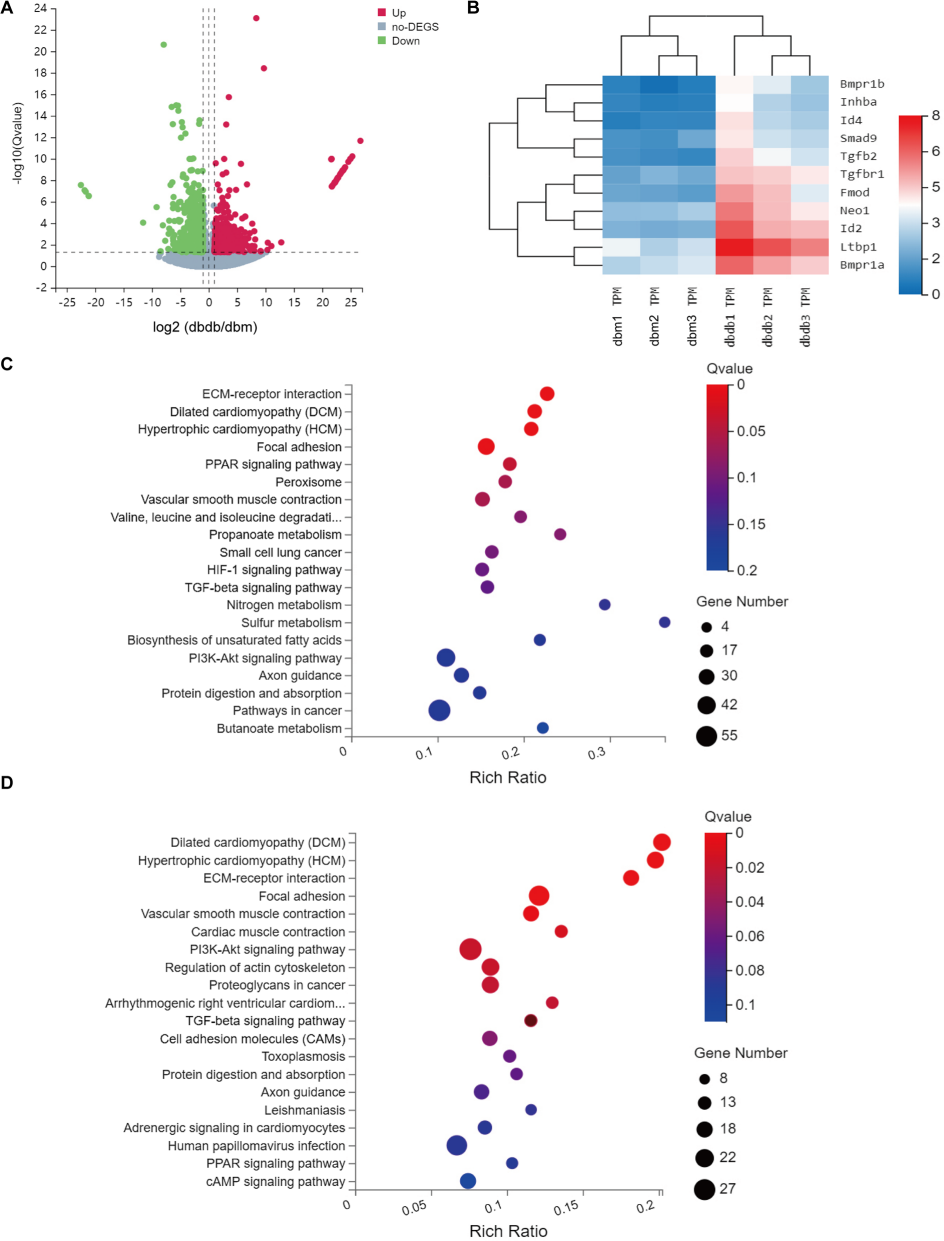
The analytical results of RNA sequencing data (**A**) The volcano plot of the differentially expressed genes of aortas isolated from *db/db* or *db/m* mice (n = 3 for each group). 1049 up-regulated (red) and 1043 down-regulated (green) genes are shown. **B** The heatmap of differentially expressed genes enriched in TGF-β signaling pathway. The gene symbols are shown on the right (n = 3 for each group). **C** KEGG pathway analysis of the 2092 differentially expressed genes. **D** KEGG pathway analysis of the 1049 upregulated expressed genes

Next, to determine whether activation of TGF-β signaling pathway was a main characteristics of gene profile changes in response to type 2 diabetes, aortic tissues isolated from a widely used type 2 diabetic rodent model, *db/db* mice and their controls *db/m* mice, were performed RNA sequencing and finally 1049 up-regulated and 1043 down-regulated differentially expressed genes were screened out for further analysis (Fig. 5A, Additional file 5: Table S5). Many of genes involved in TGF-β signaling pathway were significantly upregulated in *db/db* mice (Fig. 5B). KEGG analysis clearly revealed that these 2092 genes were mainly enriched in extracellular matrix-receptor interaction, cardiomyopathy, and vascular smooth muscle contraction, implying that vascular remodeling and increased vasocontraction might exist in *db/db* mice, in which TGF-β signaling pathway might also played a role (Fig. 5C). In addition, TGF-β signaling pathway was also involved in KEGG analysis results of 1049 up-regulated genes but not in those of down-regulated genes (Fig. 5D, Additional file 1: Fig. S3, Additional files 6, 7: Tables S6, S7), indicating TGF-β signaling pathway was activated in the aortic tissues of *db/db* mice. However, we did not detect Fbn1, the gene encoding asprosin, in these differentially expressed genes (Additional file 5: Table S5), indicating that the expression of aortic asprosin would not be affected in *db/db* diabetic mice.

### Asprosin directly induces EndMT in a TGF-β-dependent manner

**Fig. 6:**
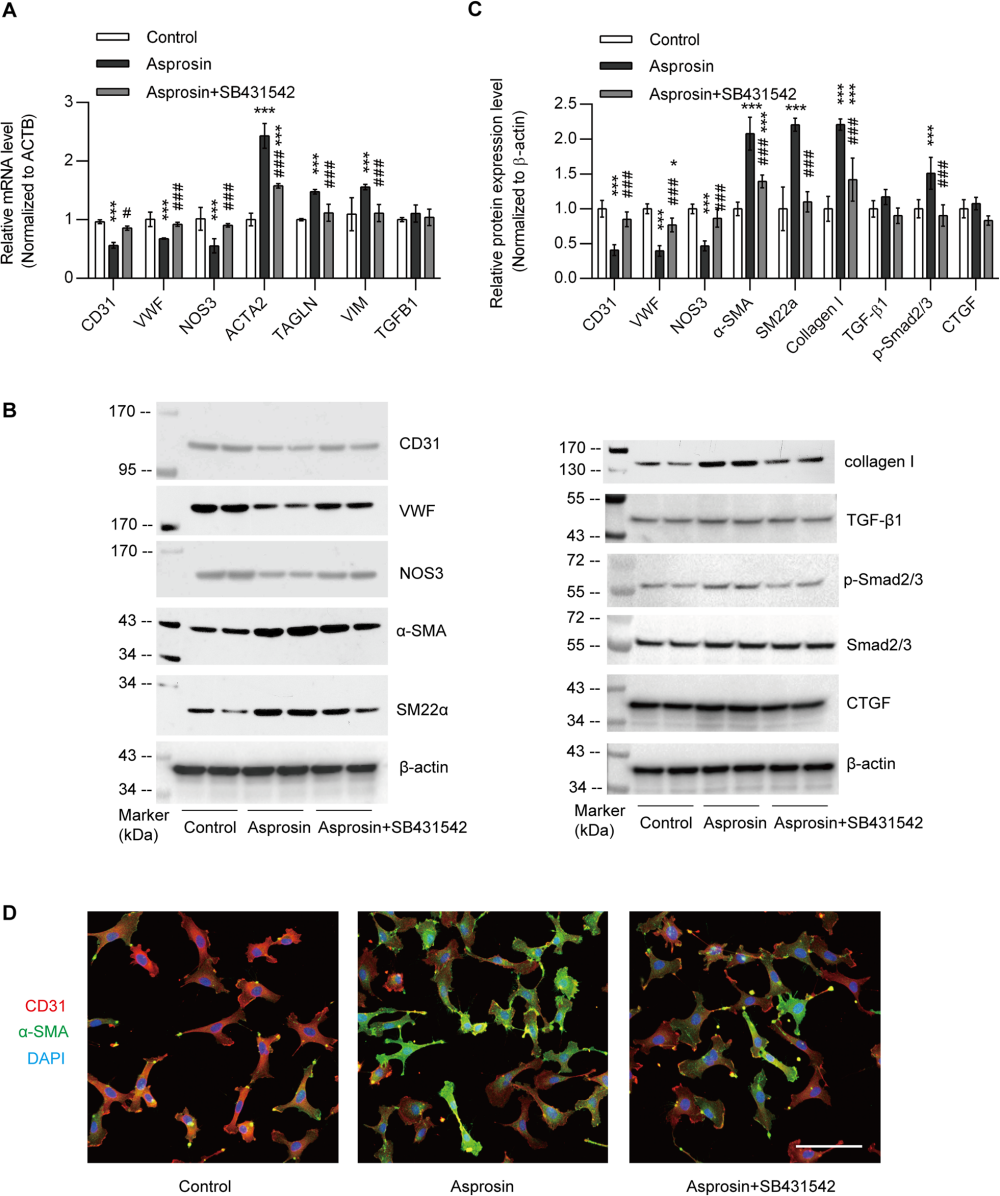
Asprosin induces EndMT in HUVECs. **A** qPCR analyses of mRNA levels for genes related to EndMT in HUVECs treated with vehicle control, 50 μmol/l asprosin, 10 μmol/l SB431542 (n = 4 for each group). The mRNA expression is normalized to ACTB (β-actin). **B**, **C** Representative western blots of indicated molecules in HUVECs. The quantitative results are shown in **C** (n = 6). Protein levels were normalized to those of β-actin. **D** Representative immunofluorescent chemical staining of CD31 and α-SMA in HUVECs. Scale bar, 100 μm. Results are expressed as mean ± s.d in **A** and **C**. *P < 0.05, ***P < 0.001, compared with control group; ^#^*P* < 0.05, ^###^*P* < 0.001, compared with asprosin group, by one-way ANOVA

To further determine whether asprosin might indeed affect vascular injury independent of diabetic status, we treated HUVECs with exogenous added asprosin protein. Treatment of HUVECs with asprosin directly induced an obvious transdifferentiating from endothelial cell to myofibroblast, namely EndMT, as evidenced by remarkable elevated expression level of a-SMA, TAGLN, collagen I, CTGF, the markers of myofibroblasts, and decreased expression of CD31, vwf, Nos3, the endothelial markers (Figs. 6A–C). In addition, immunofluorescent staining also confirmed a higher a-SMA and lower CD31 expression in response to asprosin, indicating an EndMT process (Fig. 6D). Finally, SB431542, the specific inhibitor of TGF-β signaling pathway, was added to asprosin-treated cells to determine the possible involvement of TGF-β signaling pathway in the pro-EndMT effect of asprosin. And the results clearly indicated that SB431542 remarkably erased the promotional effect of asprosin on EndMT, as all gene expression profiles that happened in response to asprosin treatment were reduced (Figs. 6A–D). These results indicate that asprosin acts as an activator of TGF-β signaling pathway to induce EndMT process in endothelial cells that might result in vascular injury in lower extremity PAD.

## Discussion

The present study confirms that asprosin is a critical adipose-tissue-derived secretory factor that facilitates the development of lower extremity PAD in patients with metabolic syndrome. Mechanistically, EndMT induced by activation of TGF-β signaling pathway at least in part mediates the promotional effect of asprosin on vascular injury that leads to collagen deposition and stiffness of vascular wall during PAD.

### The possible promotional effect of asprosin on lower extremity PAD depends on its local action on arteries

Increasing evidence have indicated that as a main feature of metabolic syndrome, obesity-related pathophysiologic changes of adipose tissue promote the cardiovascular damage in patients with metabolic disorders [23]. Indeed, previous studies also identified a broad range of adipokines that displays potent regulatory functions on cardiovascular systems, such as leptin, adiponectin and FGF21 [23]. Researchers also notice that the abnormal expression or activity of these factors caused by obesity or insulin resistance might be a critical step that leads to pathophysiological changes in cardiovascular system, possibly via promotion of inflammation, ROS generation and eNOS uncoupling [23]. Especially, emerging evidence have also revealed the association between adipokines and the development of lower extremity PAD as altered serum level of adipokines might act as independent risk factors, for instance, reduced adiponectin [24], reduced omentin-1 [25], and elevated retinol binding protein 4 (RBP4) [26]. In addition to adipokines, several circulatory factors, such as elevated fibrinogen [27], cardiac injury biomarkers N-terminal pro B-type natriuretic peptide (NT-proBNP) and high-sensitivity cardiac Troponin T (hs-cTnT) [28], are proved to be predictors of PAD. A surrogate marker of insulin resistance, triglyceride-glucose (TyG) index, is also independently associated with PAD [29]. These findings also suggest the complexity of the pathogenesis of lower extremity PAD. However, few studies can distinguish whether these markers directly act on vascular cells to cause vascular dysfunction or just a reflection of cardiovascular damage.

As a recently identified adipokine, emerging data have suggested that asprosin may exert various effects implicated in many cardio-metabolic diseases, including type 2 diabetes mellitus, polycystic ovary syndrome (PCOS), non-alcoholic fatty liver disease (NAFLD), and heart disease [30]. Clinical evidence shows that higher circulating asprosin levels display significant positive correlations to BMI, insulin resistance (IR), fasting blood glucose and triglyceride levels [31], thus most studies focus on the regulatory effects of asprosin on metabolic organs, including liver, pancreatic islets and skeletal muscles [9, 10, 32]. Accordingly, drugs that widely used to improve metabolism, such as metformin and dapagliflozin, also decreased serum asprosin levels in diabetic patients [33, 34]. However, it is unclear whether asprosin can directly regulate the function of cardiovascular system and whether this effect depends on its effect on metabolism. Unlike the previous findings in new-diagnosed diabetic patients [11, 12], we did not observe a significantly increase of asprosin level in patients with type 2 diabetes, but remarkably elevated in diabetic patients with lower extremity PAD, propably because the patients recruited in our study had already used anti-diabetic drugs that might decrease the expression of asprosin. In addition, we also revealed that plasma asprosin level was independently associated with the parameters of PAD after adjusting of multiple variables which considered to be traditional risk factor for PAD. Accordingly, in the aortas of diabetic mice, we also did not observe an increased expression of asprosin, further imply that the vascular injury in these diabetic animals might be related to higher circulatory asprosin level. These evidence all imply that the increase of asprosin might be a culprit responsible for lower extremity PAD in obesity or diabetic patients, and this effect would not be relied on its regulation on metabolism itself but depend on the local action on arteries. However, we did not manipulate the expression of asprosin in vivo to prove its direct cardiovascular effect, which is one of the limitations of this study. In addition, why the plasma level of asprosin was further elevated in patients with PAD also needs to be investigated.

### Asprosin directly leads to EndMT by activation of TGF-β signaling pathway

The transformation from endothelium to mesenchymal cells is the key step of vascular endothelial dysfunction and subsequent vascular remodeling [35]. Through EndMT, endothelial-derived mesenchymal cells lose the proper structure and function of endothelial cells and gain stem cell properties as they not only differentiate into different mesodermal cell types, but also affect underlying smooth muscle cells to synthesize collagen and accumulate lipid that led to vascular stiffness and atherosclerosis [36]. ECs have been identified as a source of mesenchymal cells within the atherosclerotic plaque as evidenced by results from lineage tracing strategies showing the presence of double-positive endothelial–mesenchymal cell populations [37, 38]. As the main regulator of EndMT, increased TGF-β signaling has also been observed to be involved in the formation of atherosclerotic plaques [39, 40]. In the present study, we observed that asprosin can promote EndMT and collagen synthesis, and the inhibitor of TGF-β pathway significantly counteracted the effect of asprosin, indicating that asprosin promotes the occurrence of EndMT by activating TGF-β pathway. In addition, RNA sequencing data on diabetic mouse arteries also confirmed the activation of TGF-β signaling pathway. Moreover, the disorder of amino acid metabolism revealed by metabolomics was the main feature of metabolism in patients with PAD, which all point to the fact that metabolic reprogramming and de novo amino acid synthesis in collagen protein production by myofibroblasts happened. These results imply that activation of TGF-β signaling pathway might account for the endothelial dysfunction that led to lower extremity PAD in diabetic patients, and increased plasma asprosin level might play a critical role in this process. However, how asprosin promotes EndMT in vivo and whether it could be recognized as a clinical diagnostic marker or intervention target for lower extremity PAD still need to be further investigated.

## Limitations

This study is a cross-sectional study with a single-center patient cohort and the sample number is relatively small. As a circulating hormone mainly secreted by white adipose tissue (WAT), asprosin itself might regulate fat distribution by reducing browning and elevating lipid deposition in adipose tissue [41]. However, in this study, we enrolled the BMI- and waist circumference- matched healthy volunteers in order to exclude the possible impact of body weight, and the distribution of adipose tissue in participants is evaluated only by the simple body fat parameters including BMI and waist circumference but not computed tomography, a golden standard for evaluation of fat distribution. Thus, a further study with better technical means might be necessary to explore the relationship between fat distribution and asprosin. In addition, it is possible that statins and hypoglycemic agents used by the recruited patients can play a role in the homeostasis of this adipokine. Therefore, recruiting newly diagnosed patients who have not been hospitalized may help to better investigating the relationship between asprosin and vascular injuries, but this may be difficult to implement as a diabetic patient with PAD often has a longer history of medication. Furthermore, it was not possible to study a relationship between aerobic exercise and the levels of this adipokine, even though aerobic exercise has been reported to decreases circulating asprosin in subjects with metabolic disorders [42].

## Conclusions

In summary, this study reveals a previously unrecognized relationship between asprosin and vascular injury in diabetic patients with lower extremity PAD and discovers the direct role of asprosin in EndMT that participates in PAD via activation of TGF-β signaling pathway. These findings imply that asprosin might be a new target for treatment and intervention of lower extremity PAD.

## Supplementary Information

**Additional file 1: Table S1**. Primers used in this study. **Figure S1.** The metabolomic analysis on serums from participants using One-way ANOVA. **Figure S2**. The metabolomic analysis on serums from participants. **Figure S3.** KEGG pathway analysis of the 1043 down-regulated expressed genes in aorta tissues from db/db mice compared to db/m mice.

**Additional file 2: Table S2.** The differentially expressed metabolites in serums from participants using One-way ANOVA.

**Additional file 3: Table S3.** The 59 differentially expressed metabolites that exist simultaneously in DM + PAD group compared to both DM and NC group.

**Additional file 4: Table S4.** The 4 differentially expressed metabolites that exist simultaneously when comparing DM + PAD group to DM group and comparing DM group to NC group.

**Additional file 5: Table S5.** All differentially expressed genes in the aorta tissues from db/db mice compared to db/m mice.

**Additional file 6: Table S6.** Upregulated expressed genes in the aorta tissues from db/db mice compared to db/m mice.

**Additional file 7: Table S7.** Downregulated expressed genes in the aorta tissues from db/db mice compared to db/m mice.

## Data Availability

The datasets used and/or analyzed for this study are available from the corresponding author upon reasonable request.

## Abbreviations

PAD: Peripheral artery disease
T2DM: Type 2 diabetes mellitus
NC: Normal control
HUVECs: Human umbilical vein endothelial cells
EndMT: Endothelial-to-mesenchymal transition
ABI: Ankle-brachial index
BMI: Body mass index
ROC: Receiver-operator characteristic
FGF21: Fibroblast growth factor 21
FMD: Flow-mediated dilation
AGEs: Advanced glycation end-products
ROS: Reactive oxygen species
NO: Nitric oxide
eNOS: Endothelial NO synthase
ECs: Endothelial cells
AUC: Area under curve
FPG: Fasting plasma glucose
HbA1c: Hemoglobin A1c
OGTT: Oral glucose tolerance test
DUS: Duplex Ultrasound
CTA: Computed tomography angiography
DSA: Digital subtraction angiography
BP: Blood pressure
TBI: Toe-brachial index
hs-CRP: High-sensitivity C-reactive protein
TC: Total cholesterol
TG: Triglyceride
HDL-C: High-density lipoprotein cholesterol
LDL-C: Low-density lipoprotein cholesterol
LC–MS: Liquid chromatography–mass spectrometry
FBS: Fetal bovine serum
α-SMA: Alpha smooth muscle actin
SBP: Systolic blood pressure
DBP: Diastolic blood pressure
ORs: Odds ratios
RBP4: Retinol binding protein 4
NT-proBNP: N-terminal pro B-type natriuretic peptide
hs-cTnT: High-sensitivity cardiac Troponin T
TyG: Triglyceride-glucose
PCOS: Polycystic ovary syndrome
NAFLD: Non-alcoholic fatty liver disease
IR: Insulin resistance
WAT: White adipose tissue
