# Supplementary material for: Asprosin induces vascular endothelial-to-mesenchymal transition in diabetic lower extremity peripheral artery disease
Source: Cardiovasc Diabetol. 2022 Feb 15;21:25. doi: 10.1186/s12933-022-01457-0 (PMC8848671; doi:10.1186/s12933-022-01457-0)
Supplement: Supplementary file 1 — Additional file 1: Table S1. Primers used in this study. Figure S1. The metabolomic analysis on serums from participants using One-way ANOVA. Figure S2. The metabolomic analysis on serums from participants. Figure S3. KEGG pathway analysis of the 1043 down-regulated expressed genes in aorta tissues from db/db mice compared to db/m mice. [file 12933_2022_1457_MOESM1_ESM.docx]

**Additional file 1**

**Asprosin induces vascular endothelial-to-mesenchymal transition in diabetic lower extremity peripheral artery disease**

Mei You, Yushuang Liu, Bowen Wang, Li Li, Hexuan Zhang, Hongbo He, Qing Zhou, Tingbing Cao, Lijuan Wang, Zhigang Zhao, Zhiming Zhu*, Peng Gao*, Zhencheng Yan*

Department of Hypertension and Endocrinology, Center for Hypertension and Metabolic Diseases, Daping Hospital, Army Medical University, Chongqing Institute of Hypertension, Chongqing 400042, China

*Correspondence to Zhencheng Yan, MD, or Peng Gao, PhD and Zhiming Zhu, MD, Department of Hypertension and Endocrinology, Center for Hypertension and Metabolic Diseases, Daping Hospital, Army Medical University, Chongqing Institute of Hypertension, 10 Chang Jiang Zhi Lu, Yuzhong District, Chongqing 400042, China. Email address: [zhuzm@yahoo.com](mailto:zhuzm@yahoo.com) (Z. Z.); [gaopengscu@aliyun.com](mailto:gaopengscu@aliyun.com) (P. G.); [zhenchengyan@sina.com.cn](mailto:zhenchengyan@sina.com.cn) (Z. Y.)

**Table S1**. Primers used for qRT-PCR.

| Gene | Primer sequence |
| --- | --- |
| CD31-fw | CCACGCCTAGCCAAAATCAC |
| CD31-rv | CATGTGGCCCCTCAGAAGAC |
| VWF-fw | CCGATGCAGCCTTTTCGGA |
| VWF-rv | TCCCCAAGATACACGGAGAGG |
| NOS3-fw | CACATGGCCTTGGACTGAA |
| NOS3-rv | CAGAGCCCTGGCCTTTTC |
| ACTA2-fw | CTATCCAGGCGGTGCTGTCTCT |
| ACTA2-rv | GCCACGCTCAGTCAGGATCTTC |
| TAGLN-fw | GCCTCAGCCCAACTTCTTACCC |
| TAGLN-rv | TTGCCGCCAAGGAGGACAGT |
| VIM-fw | GCAAAGATTCCACTTTGCGT |
| VIM-rv | GAAATTGCAGGAGGAGATGC |
| TGFB1-fw | GGCCAGATCCTGTCCAAGC |
| TGFB1-rv | GTGGGTTTCCACCATTAGCAC |
| ACTB-fw | CCTCCATCGTCCACCGCAAATG |
| ACTB-rv | TGCTGTCACCTTCACCGTTCCA |


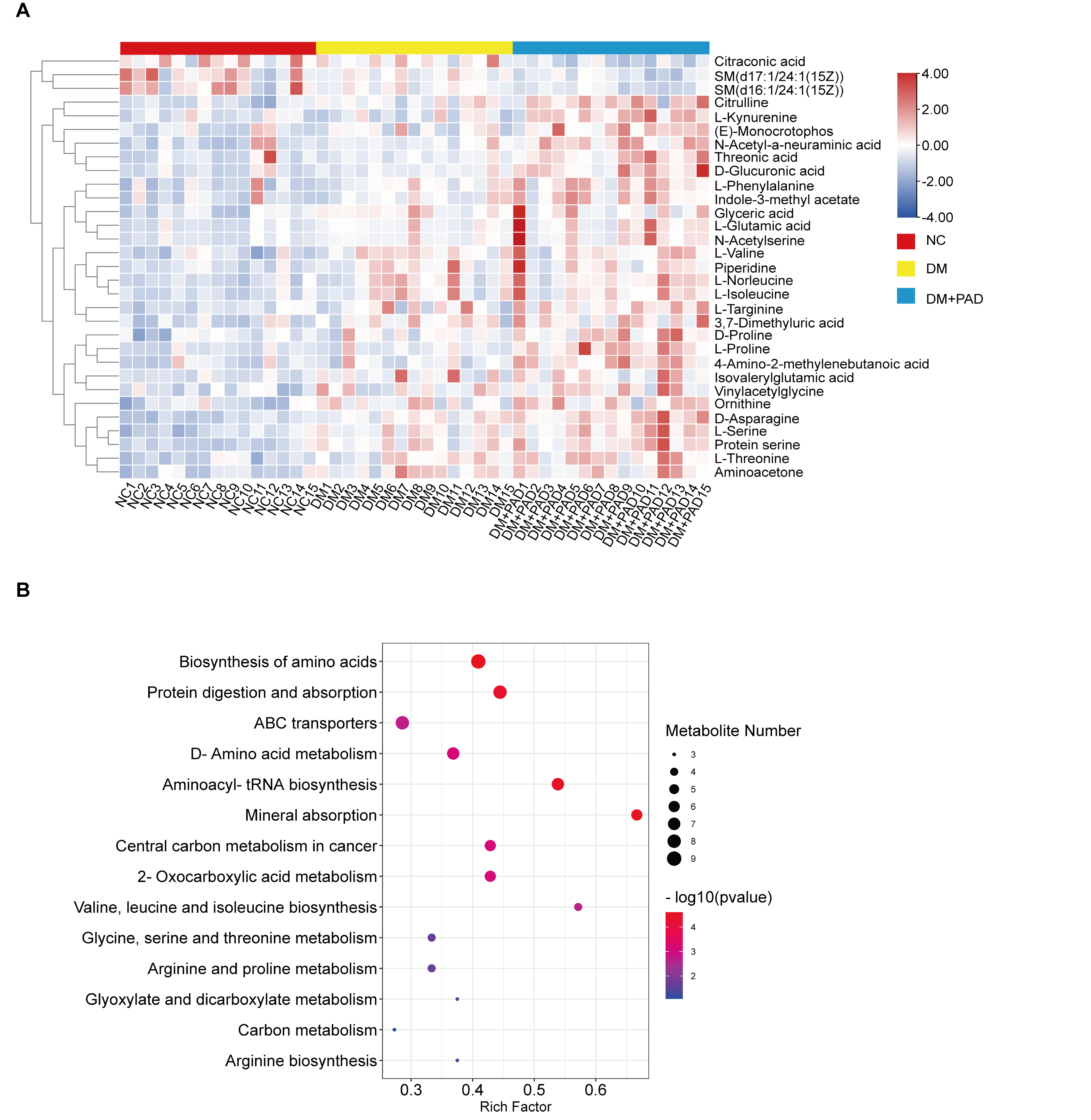


**Fig.S1** The metabolomic analysis on serums from participants using One-way ANOVA. (A) The heatmap of the differentially expressed metabolites. (C) KEGG pathway analysis of the differentially expressed metabolites.


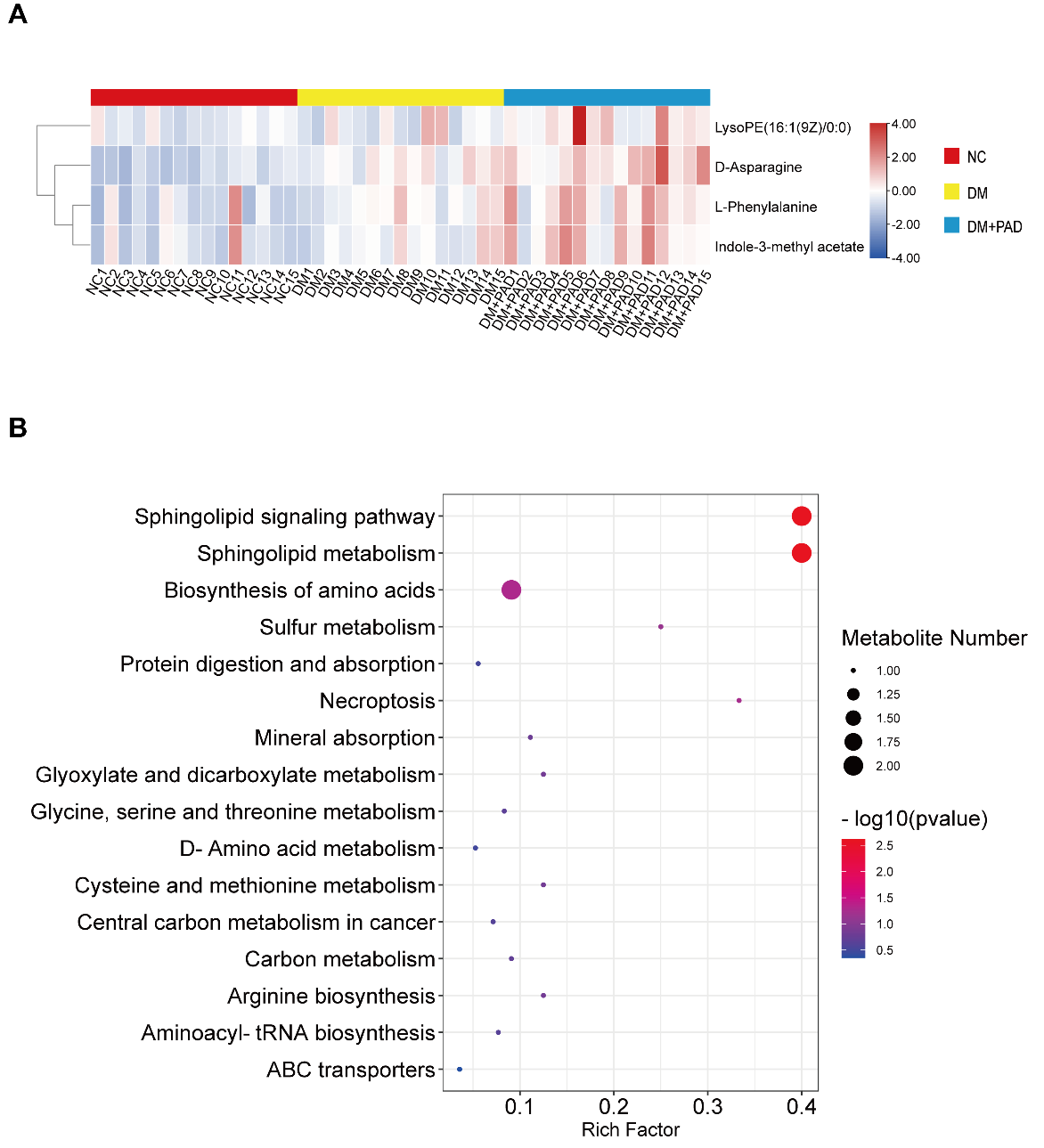


**Fig.S2** The metabolomic analysis on serums from participants (A) The heatmap of the 4 differentially expressed metabolites that exist simultaneously when comparing DM+PAD group to DM group and comparing DM group to NC group. (B) KEGG pathway analysis of the 4 differentially expressed metabolites.


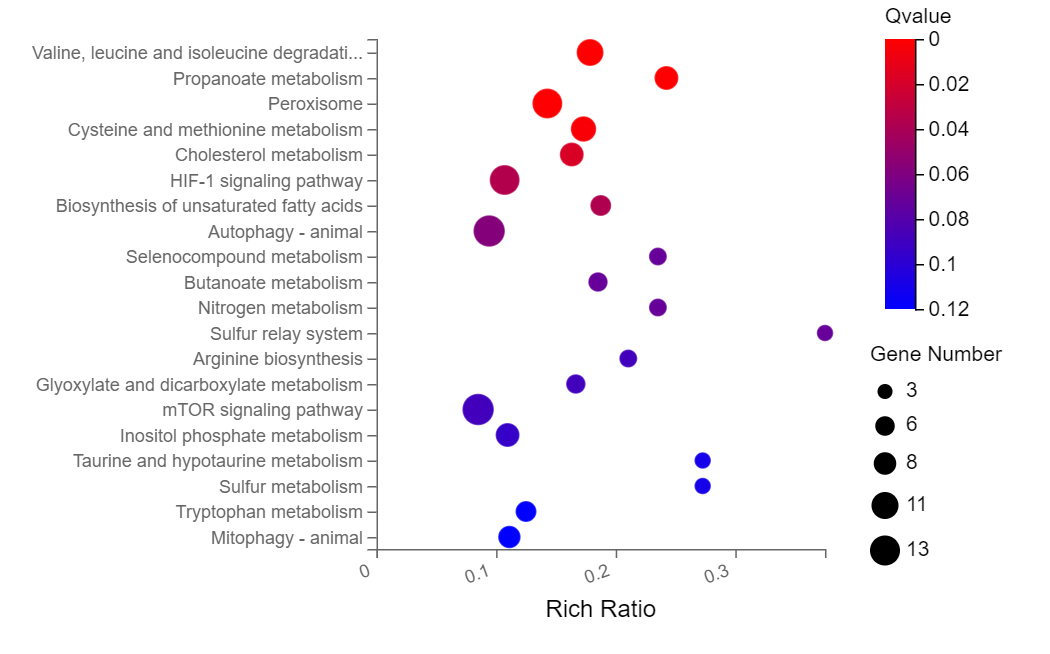


**Fig.S3** KEGG pathway analysis of the 1043 down-regulated expressed genes in aorta tissues from *db/db* mice compared to *db/m* mice.
